# Three-Dimensional Construction of a Rabbit Anterior Corneal Replacement for Lamellar Keratoplasty

**DOI:** 10.1371/journal.pone.0168084

**Published:** 2016-12-08

**Authors:** Kunpeng Pang, Liqun Du, Kai Zhang, Chenyang Dai, Chengqun Ju, Jing Zhu, Xinyi Wu

**Affiliations:** 1 Department of Ophthalmology, Qilu Hospital of Shandong University, Jinan, Shandong, China; 2 Key Laboratory of Cardiovascular Remodeling and Function Research, Chinese Ministry of Education and Chinese Ministry of Health, Qilu Hospital of Shandong University, Jinan, Shandong, China; 3 Department of Ophthalmology, the Provincial Hospital Affiliated to Shandong University, Jinan, Shandong, China; Georgetown University, UNITED STATES

## Abstract

The aim of this study was to construct a rabbit anterior corneal replacement for transplantation using acellular porcine corneal matrix (APCM) and rabbit epithelial or stromal cells. APCM was prepared from fresh porcine cornea treated with 0.5% (wt./vol.) sodium dodecyl sulfate (SDS) solution. The expanded stromal cells were first injected into APCM parallel to its surface and were cultured in a shaking culture system for 7 days to obtain the stromal construct. Next, corneal epithelial cells were cultured on the stromal construct surface for another 7 days to obtain rabbit anterior corneal lamella. The construct had a phenotype similar to that of normal cornea, with high expression of cytokeratin 3 in the epithelial cell layer and vimentin in the stromal cells. More importantly, the construct integrated well with the implanted host corneal tissue, and the implant cornea maintained transparency in the 6-month follow-up, although there was a slight haze in the central corneal area. The endothelium in the surgery cornea had a similar cell density and mosaic pattern with normal cornea as shown by confocal laser corneal microscopy, and the regenerated corneal epithelial cells on the implant surface showed a similar morphology to that of natural epithelial cells. These results demonstrate that the constructed anterior corneal replacement exhibits an excellent biological property for lamellar keratoplasty and might be a possible alternative to human corneal tissue in the future.

## Introduction

The cornea is a transparent tissue outside the eye and serves as a barrier for the protection of the eye inner tissues and is a major refractive element of the visual system [1]. Irreversible damage to the cornea can result in permanent visual loss or blindness. Next to cataracts, corneal disease is the next largest cause of vision loss, especially in developing countries [1]. According to statistics, there are more than 50 million people in the world with visual loss caused by corneal opacification resulting from disease or trauma [2].

The most successful and widely used treatment for corneal blindness is transplantation with human donor corneal tissue in several procedures such as penetrating keratoplasty [3], anterior lamellar keratoplasty (ALK) or deep ALK (DALK)[4, 5]. However, there is a severe shortage of acceptable donor corneal tissue, especially in developing countries [6]. The situation has become increasingly serious due to the increasing use of corneal laser vision corrective surgery and increase in the incidence of transmissible diseases such as HIV and hepatitis [7–9]. To solve this problem, several groups have tried to develop corneal substitutes such as tissue engineering cornea (which is very prevalent today) [10–16] and keratoprostheses [17, 18]. Presently, several types of keratoprostheses have been used in the clinic. However, they still have serious complications, including retroprosthetic membrane formation, neovascularization, infection and glaucoma [17]. Therefore, their use was limited in the clinic.

The human cornea is a transparent and multilayered tissue consisting of three layers: epithelium, stromal and endothelium, and accounts for 70% of ocular refraction [19]. Ninety percent of the corneal thickness is made up of corneal stroma (a non-renewable tissue) and will be repaired by opaque scar tissues when it is damaged [16, 20]. A valuable scaffold for corneal tissue engineering as a substitute to replace the damaged corneal tissue should have high optical clarity, toughness to withstand surgical procedures, nontoxicity and good biocompatibility [12]. Many researchers have developed several corneal substitutes similar to natural cornea using natural or synthetic polymers with corneal cells seeded within [16, 21–23]. However, these corneal replacements have been limited to in vitro applications due to the delicate mechanical properties for corneal transplantation or bad biocompatibility.

Recently, we have reported a new scaffold developed from porcine cornea named acellular porcine cornea matrix (APCM) [24–27]. The APCM has a similar optical clarity and structure of native cornea, and has the critical features of normal cornea, especially the mechanical properties. Moreover, the scaffold withstands the corneal epithelial, stromal and endothelial cell growth [24, 26]. However, we don’t know whether the bare APCM could be used for three-dimentional cornea construction, or the constructed cornea replacement from APCM have the biomechanical property of withstanding surgical procedures during the process of lamellar keratoplasty, especially the resistance of stitches. Moreover, the biofunction, biosafety and biocompatibility of the construct in vivo should be evaluated before it is used in clinic. Therefore, in this study, we constructed a rabbit anterior corneal replacement with APCM and rabbit cornea cells, and proved its excellent biofunction like natural cornea in a follow-up of 6 months after rabbit cornea transplantation.

## Materials and Methods

### Animals

Adult porcine eyes were acquired from a local slaughterhouse (Jinan Welcome Food Co., Ltd, Jinan, China) within 3 h postmortem. Thirty New Zealand white rabbits (Center for New Drugs Evaluation of Shandong University, Jinan, China) weighing 1.5–2.0 kg were used for animal transplantation and cell culture. All of the animals were treated in accordance to the ARVO (Association for Research in Vision and Ophthalmology) Statement for the Use of Animals in Ophthalmic and Vision Research. All of the animal experiments were approved by the Medical Ethics Committee of Shandong University, China. All surgeries were performed under sodium pentobarbital anesthesia, and excessive sodium pentobarbital anesthesia was used for rabbit sacrifice. All of the efforts were made to minimize suffering.

### Corneal cell culture

After rinsing three times with DMEM (Invitrogen, Waltham, MA, USA) containing 100 U/ml penicillin and 100 U/ml streptomycin (Shandong Lukang Pharmaceutical co., Ltd, Jining, China), the rabbit limbal rings were incubated at 37°C for 1 h with 2.4 U/ml Dispase II (Roche, Basel, Switzerland). The limbal epithelial sheets were separated under a dissecting microscope using two fine forceps and were digested with 0.25% Trypsin/0.02% EDTA (Sigma, Darmstadt, Germany) at 37°C for 5 min to obtain single cells.

To obtain stromal cells, 2 mm × 2 mm explants cut from the rabbit corneal stroma deprived of epithelial and endothelial cells were attached to 60-mm culture dishes to allow corneal stromal cell growth. A 1:1 mixture of Dulbecco’s minimal essential medium and Ham’s F12 medium (Invitrogen, Waltham, MA, USA), supplemented with 10% fetal bovine serum (Invitrogen, Waltham, MA, USA), 100 U/ml penicillin and 100 U/ml streptomycin was used as a growth medium for all the cells. All of the cultures were maintained under standard conditions at 37°C in a humidified atmosphere containing 5% CO_2_.

### Preparation of APCM

The method for the decellularization of porcine cornea was used as reported previously [24]. Briefly, fresh porcine corneas were immersed in a 0.5% (wt./vol.) sodium dodecyl sulfate (SDS, Sigma, Darmstadt, Germany) solution with a solvent/tissue mass ratio of 20:1 (vol./wt.), shaken for 24 h at 4°C to remove the hereditary materials, and then rinsed 8 times in sterile PBS for 16 h to obtain APCMs. Next, the APCM was washed 3 times in sterile phosphate-buffered saline (PBS, Beyotime, Nantong, China) supplemented with 200 U/ml penicillin and 200 U/ml streptomycin for 3 h, freeze-dried at -20°C for 12 h, air-dried at room temperature for 3 h in a biological safety cabinet, and stored at -20°C before use.

### Construction of anterior corneal replacement with APCM

**Fig 1:**
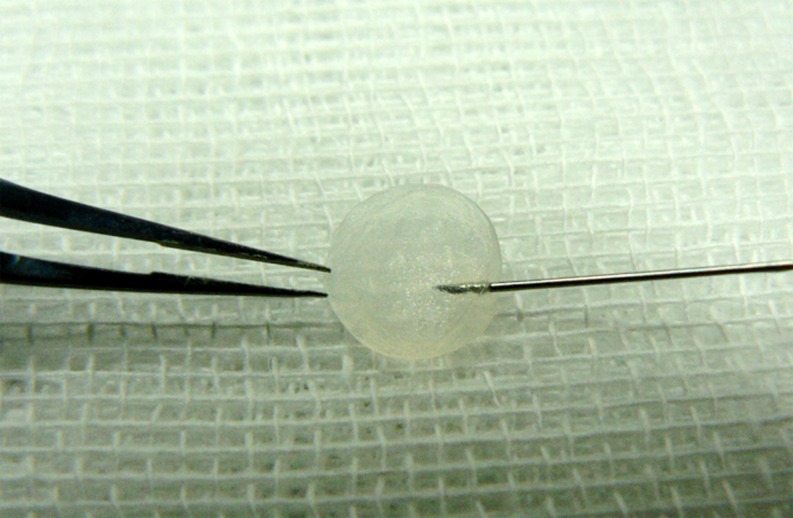
Representative image of stromal cell injection.

Before cell seeding, a thin APCM lamella (1 mm in thickness, 8 mm in diameter) containing Bowman’s membrane prepared using trephine and a scalpel under a dissecting microscope was soaked in culture medium at 37°C for 24 h. Parallel to the surface of the scaffold, a 1-ml cell suspension (5×10^5^ cells/ml) of rabbit corneal stromal cells from passage 3 was injected into the prepared APCM with a 1-ml insulin syringe (Fig 1) and was cultured on an orbital shaker for 7 days, at a rotation rate of 15 ~ 20 rpm. Next, the rabbit corneal epithelial cells from passage 1 were gently seeded onto the surface of the reconstructed stroma with a cell density of 5 × 10^3^/mm^2^ and cultured for next 7 days. All of the constructs were cultured at 37°C in a humidified atmosphere containing 5% CO_2_. The culture medium was exchanged every two days.

### Implantation

The right eye of each New Zealand white rabbit was used for constructed anterior corneal lamellar keratoplasty. After the surgical eyes were treated with antibiotic for three days, the rabbits were anesthetized through the ear veins using 3% sodium pentobarbital (1 ml/kg). Next, 6.5-mm-diameter, half a cornea in thickness plant beds in the cornea of rabbits were made by trephine, and then the prepared constructs were implanted on it with 10–0 nylon sutures. Subsequently, the surgical eyes were covered with amniotic membranes. Subconjunctival injection of gentamicin and dexamethasone was used every two days. Eye drops containing tobramycin and dexamethasone were used 3 times each day. Follow-up clinical examinations included slit-lamp examination to evaluate the hyperemia of the conjunctiva, corneal optical clarity, neo-vascularization, and degradation of the grafts. After 1 w, 2 w, 4 w, 3 m and 6 m following surgery, the rabbits were euthanized, respectively, and were subjected to confocal laser corneal microscopy and corneal histology examination.

### Microscopy and histology

Fresh porcine corneas, APCMs, constructed anterior corneal replacements and specimens taken from implanted rabbit corneas (n = 3, respectively) were fixed in 4% formaldehyde and were embedded in paraffin. Next, 4-μm sections were stained with H&E and were viewed under a light microscope.

Before scanning electron microscopy examination, the samples were fixed in cacodylate-buffered 3% glutaraldehyde and were postfixed for 1 h in 1% osmium tetroxide. Next, the samples were gradually dehydrated by a graded ethanol series (50%, 70%, 95%, 100%), critical-point dried, and gold sputter-coated according to routine procedures[28], followed by viewing under a scanning electron microscope (SEM, S-570, Hitachi, Japan).

### Immunohistochemistry staining

Immunohistochemical staining was performed according to a previously reported method [29] to evaluate cytokeratin 3 (CK3), a marker of corneal epithelial cells [30], and vimentin, a marker of corneal stromal cells [31]. In brief, after paraffin removal and microwave-treated antigen retrieval, the endogenous peroxidase activity of the sections were quenched by treatment with 0.3% H_2_O_2_ in PBS, and then the non-specific sites were blocked with 5% goat serum. CK3 (1:200) mAb (Abcam, San Francisco, CA, USA) or vimentin (1:100) mAb (Abcam, San Francisco, CA, USA) was applied and incubated at 4°C overnight, followed by incubation with peroxidase-conjugated Affinipure goat anti-mouse secondary antibody (Zhongshan Goldbridge Biotechnology Co., LTD, Beijing, China) at 37°C for 30 min. Finally, the specimens were incubated with 3, 3’- diaminobenzidine (DAB) peroxidase substrate to give a brown stain and counterstain with hematoxylin.

## Results

### Characterization of the APCM and constructed anterior cornea replacement

**Fig 2:**
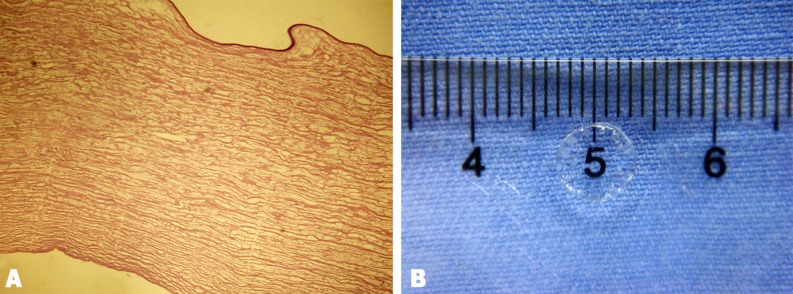
Representative image and histological characteristics of the APCM. H&E (A) staining showed that the cells were completely removed. The scaffold was transparent when soaked in 100% sterile glycerol (B).

APCM was transparent after being soaked in sterile glycerol for 30 min (Fig 2B). No visible cells or cell nuclear materials were reserved in the scaffold (Fig 2A). The scaffold had the key features of the cornea such as optical clarity, toughness to withstand surgical procedures, and good biocompatibility, as reported previously [24, 27].

**Fig 3:**
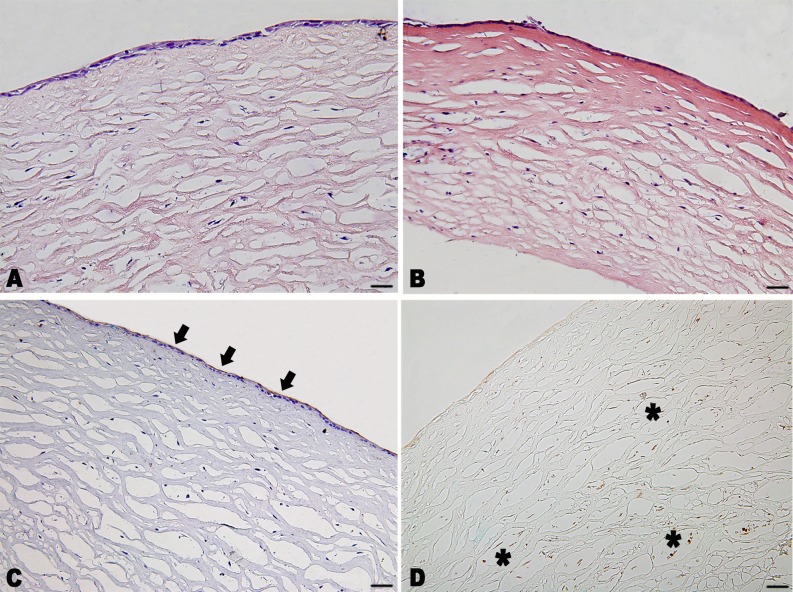
Phenotype and histological sections of the constructed rabbit anterior cornea replacement. (A) H&E staining showed two or three layers of epithelial cells formed on the scaffold with a relatively uniform cell distribution in the matrix. (B) H&E staining of the construct soaked in 100% sterile glycerol for 5 minutes. (C) Immunohistochemistry staining for cytokeratin 3 (brown, black arrow) counterstained with hematoxylin (blue), and for vimentin (brown, asterisk) without hematoxylin staining. Scale bar: 50 μm.

The phenotype of the construct was similar to that of normal rabbit corneas. Two or three layers of epithelial cells with high expression of CK3 formed on top of the stromal substitutes after 7 days of submerged culture (Fig 3A, 3B, and 3C). Rabbit cornea stromal cells grew with a relative uniform cell distribution in the matrix with the expression of vimentin, as demonstrated by immunohistochemical staining (Fig 3A, 3B and 3D).

### Implantation and clinical evaluation

**Fig 4:**
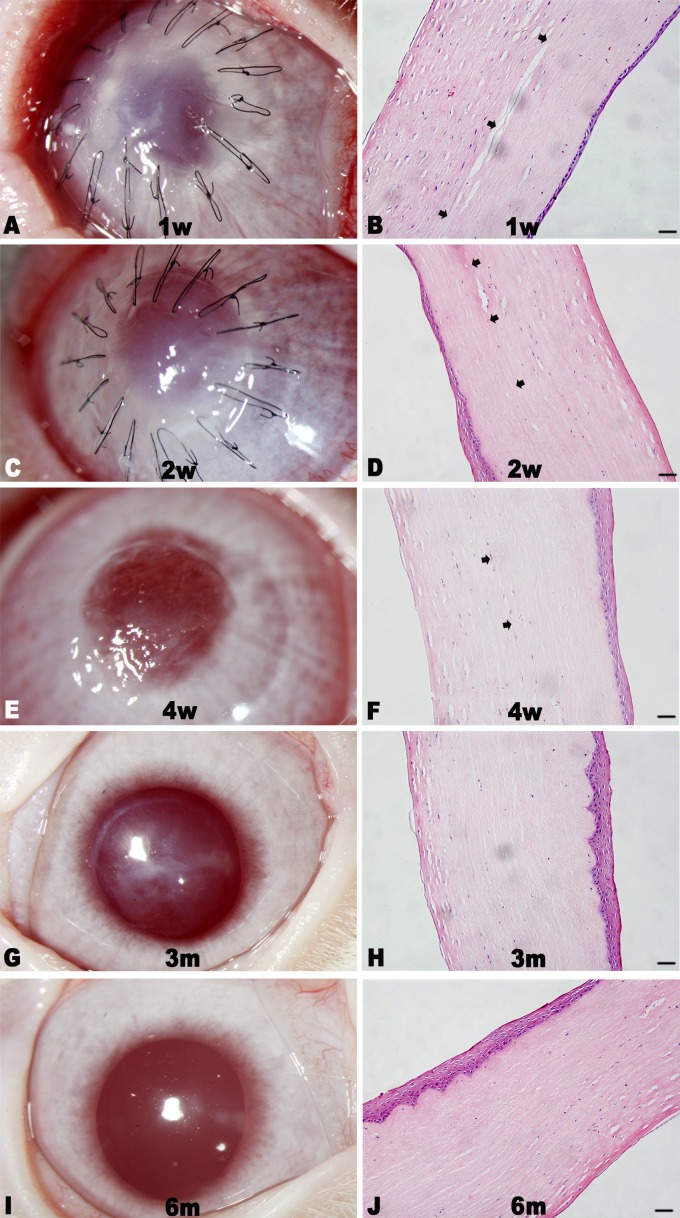
Representative images and histological sections of implanted corneas postoperative 6 months. The implanted corneas gradually cleared with no apparent neovascularization (A, C, E, G, and I), and the constructs integrated well with the host cornea tissues with regular collagen arrangement as shown by H&E staining (B, D, F, H, and J). The boundary between the implant and host tissue was clear at the 4-week follow-up (B, D, and F, black arrows), and became indistinct 3 months (H) and 6 months (J) after surgery. Scale bar: 50 μm.

**Fig 5:**
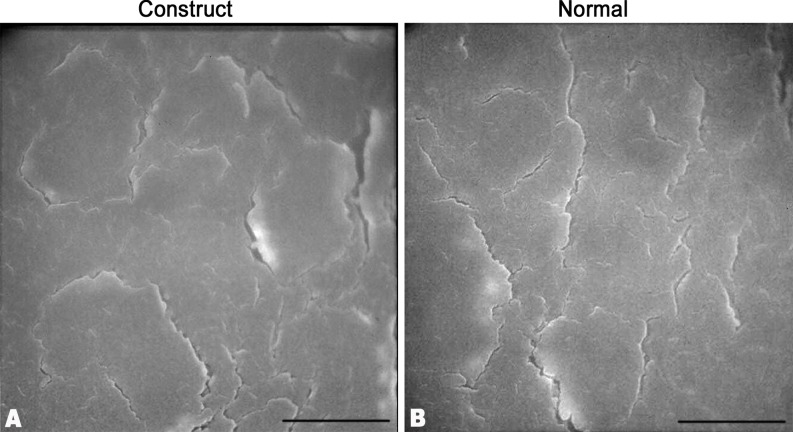
Ultrastructure of the epithelial cells of implanted cornea and normal cornea. The regenerated corneal epithelial cells on the implant surface showed a similar morphology to that of natural corneal epithelial cells. Scale bar: 10 μm.

**Fig 6:**
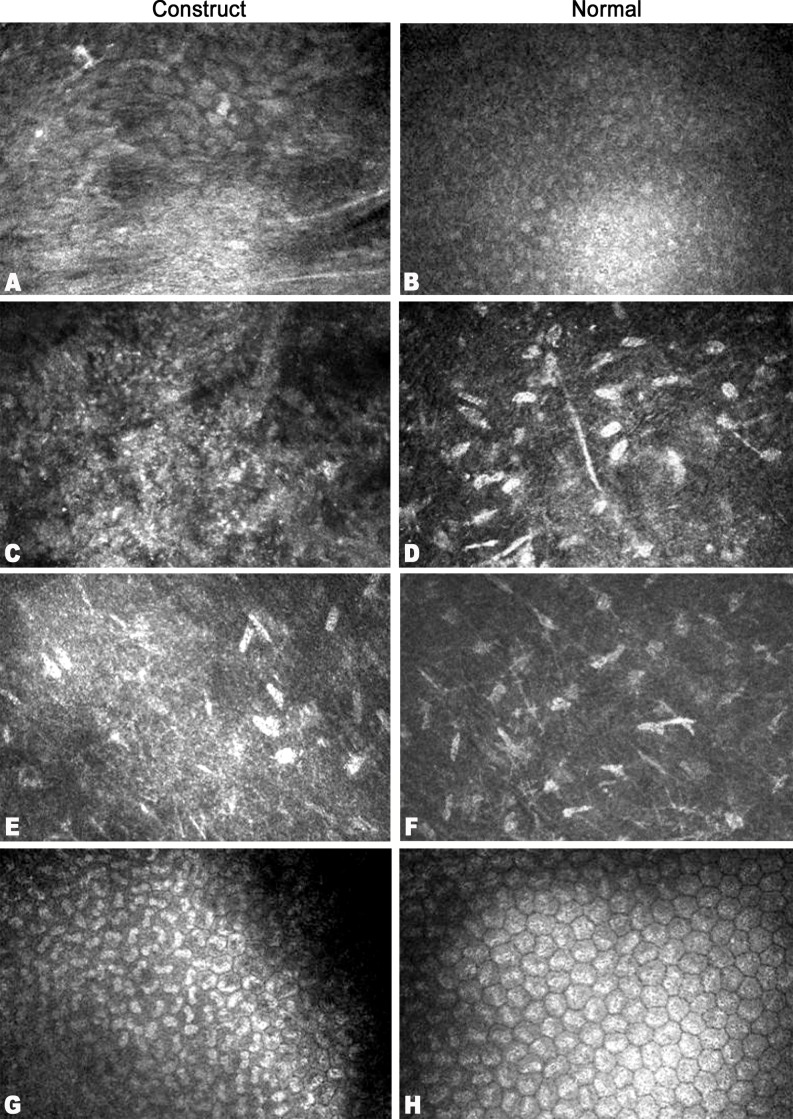
Confocal laser corneal microscopy images of corneal features in a healthy, unoperated subject, alongside those of operated corneas, at 6 months after construct implantation. Regenerated corneal epithelial cells on the implant surface (A), and intact epithelium of the normal cornea (B). Anterior stromal cell nuclei (C, D) and posterior stromal cells (E, F) were present, with no Langerhans cells or any other typical inflammatory cells immersed within. The endothelium (G, H) in all corneas exhibited a characteristic mosaic pattern.

The sutures were removed after 4 weeks of surgery. At 1 week after surgery, the amniotic membrane of the surgery eye was lost. The central cornea of the implanted eye was a little cloudy without neovascularization (Fig 4A). However, the iris texture was clear, and there was no serious conjunctiva edema and congestion appeared. The construct was different from recipient cornea tissue with few cells distributed in the stroma and 1~2 epithelial-like cell layers formed on the surface as shown by histology examination (Fig 4B). The transparency of the surgery cornea was improved with no invasion of neovascularization (Fig 4C) at 2 weeks. However, discrete focal areas of haze appeared and remained, even at 6 months (Fig 4E, 4G and 4I). Good corneal transparency at 6 months was demonstrated by the clear visualization of the iris texture through the central cornea, and a normal stratification epithelium was formed on the surface of implanted cornea with stable attachment (Fig 4H and 4J) and a similar morphology to that of natural epithelial cells (Fig 5). Moreover, the construct with stromal cells distributed in it was well integrated within the host corneas. The endothelium in the surgery cornea had a similar cell density and mosaic pattern with normal cornea as shown by confocal laser corneal microscopy (Fig 6).

## Discussion

The cornea is transparent, tough, and susceptible to diseases or trauma that can result in blindness [1, 2]. The cornea epithelium comprising 4~5 epithelial cell layers on the anterior surface of stroma is the first defensive barrier of the cornea [32]. The endothelium attached to the posterior surface of the stroma is formed by one regular endothelial cell layer, and has a dehydration pump function that maintains the transparency of the cornea [33]. Corneal stroma is a non-renewable tissue composed of approximately 250~300 collagen layers derived from a highly organized array of collagen I filaments with keratocytes dispersed for extracellular matrix maintaining [34–36]. It provides most of the structural framework of the cornea and composes approximately 80% to 90% of its thickness [32]. The special collagen network in the corneal stroma accounts for the cornea’s stability, mechanical strength and transparency [37]. Therefore, finding an ideal scaffold with good biocompatibility, high optical clarity, toughness to withstand surgical procedures, and non-immunogenic properties for tissue-engineered cornea is most important. We previously developed a scaffold named APCM from natural porcine cornea using a decellular technique [24, 27, 38]. The APCM exhibited good optical clarity, the toughness to withstand surgical procedures, and biocompatibility. In addition, the scaffold supported cell differentiation, proliferation and migration [24].

APCM preserved the three-dimensional structure of the natural cornea stroma and consist of the major molecules, including collagen type I and IV, laminin, and fibronectin, which are very important for cell differentiation, proliferation and migration [39]. Until now, there have been no synthetic scaffolds that can perfectly mimic the structure of the natural cornea stroma. More importantly, the porcine cornea has an abundant source and can be acquired easier and cheaper than synthetic scaffolds. Deep lamellar keratoplasty (DLK) is a safe alternative to penetrating keratoplasty (PK) for common diseases such as keratoconus due to the similar corrected visual acuity, refractive results, and complication rates between DLK and PK[5, 40, 41]. Moreover, the risk of endothelial rejection can be avoided[42]. Thus, we developed a rabbit anterior cornea replacement containing epithelium and part of the stroma with APCM to achieve this goal.

Reconstruction of an intact, healthy, stratified epithelial layer over a cornea substitute for transplantation is essential as a defense against infection of the cornea. In our study, two or three layers of epithelial cells quickly formed on the surface of the APCM with a tight junction in 7 days. Although there were no typical column-shaped cells, similar to natural corneal epithelium, at the bottom of the constructed epithelial layer because the construct should be put into a shaking culture environment for stromal cells growth that was inadaptable to air-lift cultural models [21], this result could provide proof that APCM supports corneal epithelial cell growth. An optimized three-dimensional culture strategy should be developed in the future.

A valuable scaffold should have a good pore structure, key cues for the induction of cell differentiation or proliferation, a valid path for the nutrient substance and waste exchange, which is very important for cell metabolism, to support stromal cell growth [20]. In our study, rabbit stromal cells grew in the matrix with a relative uniform cell distribution and exhibited good survival and rapid proliferation[24]. APCM preserved the structure of the natural cornea including the pore and molecules that can support stromal cells growing in it. More importantly, the shaking culture strategy could give a dynamic system for waste exchange that might improve cell growth. This might be a valid culture system for three-dimensional culture or organ culture.

The biomechanical property is a critical factor for cornea tissue engineering. The rabbit anterior cornea replacement constructed from APCM had the biomechanical property of withstanding surgical procedures such as the resistance of stitches. In the six-month follow-up period of lamellar keratoplasty, all of the implanted corneas were free of major complications such as stromal edema, neovascularization, or inflammation, and there were no symptoms of rejection or corneal ectasia within the implant cornea. Moreover, the cornea remained transparent with a slight haze at 6 months that was demonstrated by the clear visualization of the iris texture. Although there were some epithelium defects on the cornea in the early period of the lamellar keratoplasty due to the operative procedure and tight retaining sutures, normal corneal epithelium quickly covered the surface of the transplant after 3 weeks. Thus, a revised surgical method should be used in the future to improve the situation.

At six months after surgery, although the cornea was transparent, the corneal surface seemed irregular and with a slight haze in the implant that might reduce the vision in patients with implants if it was used in the clinic. In natural cornea stroma, there are quiescent stromal cells distributed in it responsible for collagen fibers regulation and stroma transparence maintenance. They are also named keratocytes [43]. However, with the microenvironment change, such as corneal injures, keratocytes would be lead to apoptosis or transformed to fibroblasts or myofibroblasts that can scatter light, produce corneal haze and change the collagen ingredients in the stromal extracellular matrix [44]. In this study, in order to make sure that the epithelial cells and stromal cells both grew well in the APCM using the three-dimentional organ culture system, the serum was used in it. The serum contains complicated growth factors that could turn keratocytes into fibroblasts or myofibroblasts. This might explain the irregular cornea surface or haze formation in the transplant cornea. However, the transplanted cornea was kept transparence in 6 months follow-up after transplantation. Moreover, the degree of haze and surface irregularity diminished gradually in a long term observation. Therefore, the construct in this study might have the potential for ocular surface construction and visual acuity improvement suffered with corneal ulcer, corneal leucoma or serious keratoconus. Next, the culture system should be improved to solve this problem such as the use of a serum-free culture system.

Despite the need to improve the three-dimensional culture system and refine the surgical technique for optimum visual outcome, the constructs in this study were transparent while exhibiting good biocompatibility, biosafety and regenerative features that would enable their use as a valuable substitute for human donor cornea tissue for transplantations. The constructs were quickly epithelialized, populated with cells in the stroma, and integrated into host tissue. Additionally, the implants had no detriments to the endothelium as demonstrated by in vivo corneal confocal microscopy. Although the study in the clinic should be carried out to further determine the full potential of the cornea substitutes to relieve the tension of donor cornea tissue and cornea blindness, our results demonstrate that the constructed anterior corneal replacement might be a possible alternative to human cornea tissue for LK or DLK when the endothelium is uncompromised.

## References

[1] Fagerholm P, Lagali NS, Merrett K, Jackson WB, Munger R, Liu Y, et al A biosynthetic alternative to human donor tissue for inducing corneal regeneration: 24-month follow-up of a phase 1 clinical study. Science translational medicine. 2010;2(46):46ra61 10.1126/scitranslmed.3001022 20739681

[2] Whitcher JP, Srinivasan M, Upadhyay MP. Corneal blindness: a global perspective. Bulletin of the World Health Organization. 2001;79(3):214–21. PubMed Central PMCID: PMC2566379. 11285665 PMC2566379

[3] Matthaei M, Sandhaeger H, Hermel M, Adler W, Jun AS, Cursiefen C, et al Changing Indications in Penetrating Keratoplasty: A Systematic Review of 34 Years of Global Reporting. Transplantation. 2016. 10.1097/TP.0000000000001281 27336399

[4] Goweida MB. Intraoperative review of different bubble types formed during pneumodissection (big-bubble) deep anterior lamellar keratoplasty. Cornea. 2015;34(6):621–4. 10.1097/ICO.0000000000000407 25909235

[5] Arnalich-Montiel F, Alio Del Barrio JL, Alio JL. Corneal surgery in keratoconus: which type, which technique, which outcomes? Eye and vision. 2016;3:2 PubMed Central PMCID: PMC4716637. 10.1186/s40662-016-0033-y 26783544 PMC4716637

[6] Lagali N, Griffith M, Fagerholm P, Merrett K, Huynh M, Munger R. Innervation of tissue-engineered recombinant human collagen-based corneal substitutes: a comparative in vivo confocal microscopy study. Investigative ophthalmology & visual science. 2008;49(9):3895–902. 18408185 10.1167/iovs.07-1354

[7] Dubord PJ, Evans GD, Macsai MS, Mannis MJ, Glasser DB, Strong DM, et al Eye banking and corneal transplantation communicable adverse incidents: current status and project NOTIFY. Cornea. 2013;32(8):1155–66. 10.1097/ICO.0b013e31828f9d64 23676781

[8] Hogan RN, Brown P, Heck E, Cavanagh HD. Risk of prion disease transmission from ocular donor tissue transplantation. Cornea. 1999;18(1):2–11. 9894930

[9] Armitage WJ, Tullo AB, Ironside JW. Risk of Creutzfeldt-Jakob disease transmission by ocular surgery and tissue transplantation. Eye. 2009;23(10):1926–30. 10.1038/eye.2008.381 19136921

[10] Carlsson DJ, Li F, Shimmura S, Griffith M. Bioengineered corneas: how close are we? Current opinion in ophthalmology. 2003;14(4):192–7. 12888716 10.1097/00055735-200308000-00004

[11] McLaughlin CR, Fagerholm P, Muzakare L, Lagali N, Forrester JV, Kuffova L, et al Regeneration of corneal cells and nerves in an implanted collagen corneal substitute. Cornea. 2008;27(5):580–9. 10.1097/ICO.0b013e3181658408 18520509

[12] Liu Y, Gan L, Carlsson DJ, Fagerholm P, Lagali N, Watsky MA, et al A simple, cross-linked collagen tissue substitute for corneal implantation. Investigative ophthalmology & visual science. 2006;47(5):1869–75. 16638993 10.1167/iovs.05-1339

[13] Kim do K, Sim BR, Khang G. Nature-Derived Aloe Vera Gel Blended Silk Fibroin Film Scaffolds for Cornea Endothelial Cell Regeneration and Transplantation. ACS applied materials & interfaces. 2016;8(24):15160–8. 27243449 10.1021/acsami.6b04901

[14] Rafat M, Xeroudaki M, Koulikovska M, Sherrell P, Groth F, Fagerholm P, et al Composite core-and-skirt collagen hydrogels with differential degradation for corneal therapeutic applications. Biomaterials. 2016;83:142–55. 10.1016/j.biomaterials.2016.01.004 26773670

[15] Abidin FZ, Gouveia RM, Connon CJ. Application of retinoic acid improves form and function of tissue engineered corneal construct. Organogenesis. 2015;11(3):122–36. PubMed Central PMCID: PMC4879898. 10.1080/15476278.2015.1093267 26496651 PMC4879898

[16] Griffith M, Osborne R, Munger R, Xiong X, Doillon CJ, Laycock NL, et al Functional human corneal equivalents constructed from cell lines. Science. 1999;286(5447):2169–72. 10591651 10.1126/science.286.5447.2169

[17] Salvador-Culla B, Kolovou PE. Keratoprosthesis: A Review of Recent Advances in the Field. Journal of functional biomaterials. 2016;7(2). 10.3390/jfb7020013 PMC4932470 27213461

[18] Duignan ES, Ni Dhubhghaill S, Malone C, Power W. Long-term visual acuity, retention and complications observed with the type-I and type-II Boston keratoprostheses in an Irish population. Br J Ophthalmol. 2015. 10.1136/bjophthalmol-2015-307443 26628625

[19] Reichl S, Muller-Goymann CC. The use of a porcine organotypic cornea construct for permeation studies from formulations containing befunolol hydrochloride. International journal of pharmaceutics. 2003;250(1):191–201. 12480285 10.1016/s0378-5173(02)00541-0

[20] Alaminos M, Del Carmen Sanchez-Quevedo M, Munoz-Avila JI, Serrano D, Medialdea S, Carreras I, et al Construction of a complete rabbit cornea substitute using a fibrin-agarose scaffold. Investigative ophthalmology & visual science. 2006;47(8):3311–7. 16877396 10.1167/iovs.05-1647

[21] Minami Y, Sugihara H, Oono S. Reconstruction of cornea in three-dimensional collagen gel matrix culture. Investigative ophthalmology & visual science. 1993;34(7):2316–24. 7685009

[22] Nishida K. Tissue engineering of the cornea. Cornea. 2003;22(7 Suppl):S28–34. 14703705 10.1097/00003226-200310001-00005

[23] Couture C, Zaniolo K, Carrier P, Lake J, Patenaude J, Germain L, et al The tissue-engineered human cornea as a model to study expression of matrix metalloproteinases during corneal wound healing. Biomaterials. 2016;78:86–101. 10.1016/j.biomaterials.2015.11.006 26686051

[24] Pang K, Du L, Wu X. A rabbit anterior cornea replacement derived from acellular porcine cornea matrix, epithelial cells and keratocytes. Biomaterials. 2010;31(28):7257–65. 10.1016/j.biomaterials.2010.05.066 20598368

[25] Zhu J, Zhang K, Sun Y, Gao X, Li Y, Chen Z, et al Reconstruction of functional ocular surface by acellular porcine cornea matrix scaffold and limbal stem cells derived from human embryonic stem cells. Tissue engineering Part A. 2013;19(21–22):2412–25. 10.1089/ten.TEA.2013.0097 23675636

[26] Ju C, Zhang K, Wu X. Derivation of corneal endothelial cell-like cells from rat neural crest cells in vitro. PloS one. 2012;7(7):e42378 PubMed Central PMCID: PMC3409168. 10.1371/journal.pone.0042378 22860120 PMC3409168

[27] Du L, Wu X, Pang K, Yang Y. Histological evaluation and biomechanical characterisation of an acellular porcine cornea scaffold. Br J Ophthalmol. 2011;95(3):410–4. 10.1136/bjo.2008.142539 20956275

[28] Sanchez-Quevedo MC, Moreu G, Campos A, Garcia JM, Gonzalez-Jaranay M. Regional differences in cell surface patterns in normal human sulcular epithelium. Histology and histopathology. 1994;9(1):149–53. 8003810

[29] Yoshino K, Tseng SC, Pflugfelder SC. Substrate modulation of morphology, growth, and tear protein production by cultured human lacrimal gland epithelial cells. Experimental cell research. 1995;220(1):138–51. 10.1006/excr.1995.1300 7664830

[30] Schermer A, Galvin S, Sun TT. Differentiation-related expression of a major 64K corneal keratin in vivo and in culture suggests limbal location of corneal epithelial stem cells. The Journal of cell biology. 1986;103(1):49–62. PubMed Central PMCID: PMC2113783. 2424919 10.1083/jcb.103.1.49 PMC2113783

[31] Chen CC, Chang JH, Lee JB, Javier J, Azar DT. Human corneal epithelial cell viability and morphology after dilute alcohol exposure. Investigative ophthalmology & visual science. 2002;43(8):2593–602. 12147590

[32] de Araujo AL, Gomes JA. Corneal stem cells and tissue engineering: Current advances and future perspectives. World journal of stem cells. 2015;7(5):806–14. PubMed Central PMCID: PMC4478627. 10.4252/wjsc.v7.i5.806 26131311 PMC4478627

[33] Zavala J, Lopez Jaime GR, Rodriguez Barrientos CA, Valdez-Garcia J. Corneal endothelium: developmental strategies for regeneration. Eye. 2013;27(5):579–88. PubMed Central PMCID: PMC3650267. 10.1038/eye.2013.15 23470788 PMC3650267

[34] Meller D, Peters K, Meller K. Human cornea and sclera studied by atomic force microscopy. Cell and tissue research. 1997;288(1):111–8. 9042778 10.1007/s004410050798

[35] Freegard TJ. The physical basis of transparency of the normal cornea. Eye. 1997;11 (Pt 4):465–71. 9425408 10.1038/eye.1997.127

[36] DelMonte DW, Kim T. Anatomy and physiology of the cornea. Journal of cataract and refractive surgery. 2011;37(3):588–98. 10.1016/j.jcrs.2010.12.037 21333881

[37] Boote C, Dennis S, Newton RH, Puri H, Meek KM. Collagen fibrils appear more closely packed in the prepupillary cornea: optical and biomechanical implications. Investigative ophthalmology & visual science. 2003;44(7):2941–8. 12824235 10.1167/iovs.03-0131

[38] Ju C, Gao L, Wu X, Pang K. A human corneal endothelium equivalent constructed with acellular porcine corneal matrix. The Indian journal of medical research. 2012;135(6):887–94. PubMed Central PMCID: PMC3410216. 22825608 PMC3410216

[39] Ponce Marquez S, Martinez VS, McIntosh Ambrose W, Wang J, Gantxegui NG, Schein O, et al Decellularization of bovine corneas for tissue engineering applications. Acta biomaterialia. 2009;5(6):1839–47. 10.1016/j.actbio.2009.02.011 19286434

[40] Razmjoo H, Ghoreishi SM, Ashtari A, Ghafouri I, Mamousi B. Comparison the post operative refractive errors in same size corneal transplantation through deep lamellar keratoplasty and penetrating keratoplasty methods after sutures removing in keratoconus patients. Advanced biomedical research. 2016;5:39 PubMed Central PMCID: PMC4815524. 10.4103/2277-9175.178784 27099852 PMC4815524

[41] Watson SL, Ramsay A, Dart JK, Bunce C, Craig E. Comparison of deep lamellar keratoplasty and penetrating keratoplasty in patients with keratoconus. Ophthalmology. 2004;111(9):1676–82. 10.1016/j.ophtha.2004.02.010 15350322

[42] Terry MA. The evolution of lamellar grafting techniques over twenty-five years. Cornea. 2000;19(5):611–6. 11009313 10.1097/00003226-200009000-00006

[43] Espana EM, He H, Kawakita T, Di Pascuale MA, Raju VK, Liu CY, et al Human keratocytes cultured on amniotic membrane stroma preserve morphology and express keratocan. Investigative ophthalmology & visual science. 2003;44(12):5136–41. 14638709 10.1167/iovs.03-0484

[44] Jester JV, Budge A, Fisher S, Huang J. Corneal keratocytes: phenotypic and species differences in abundant protein expression and in vitro light-scattering. Investigative ophthalmology & visual science. 2005;46(7):2369–78. PubMed Central PMCID: PMC1853377. 15980224 10.1167/iovs.04-1225 PMC1853377

